# Thermal imaging for estimating melting point in cheese: A cost-effective alternative to rheology

**DOI:** 10.3168/jdsc.2025-0765

**Published:** 2025-05-30

**Authors:** Zeel Modi, Prafulla Salunke

**Affiliations:** Dairy and Food Science Department, Midwest Dairy Foods Research Center, South Dakota State University, Brookings, SD 57007

## Abstract

The melting characteristics of cheese play a pivotal role in determining functional performance in various applications. Measuring the meltability and melting point of cheese is a challenge and requires sophisticated equipment, a laboratory setup, and personnel training, and the cost can be prohibitive. Over the years, many tests have been developed to determine the meltability or melting point of cheese. Currently, the melting point of cheese is measured using a specialized instrument, a rheometer for dynamic stress rheology values, to calculate the transition temperature (tan δ). It measures the transition of cheese from the solid to the viscous phase as the cheese is heated. However, the test is time-consuming. A thermal infrared (IR) image camera and software analysis can be used to quickly measure cheese's meltability and melting point. The study was designed to develop a method to estimate the melting point of cheese using a thermal imaging camera, providing a more practical and cost-effective alternative to conventional rheological analysis. Commercial samples of cheeses, including natural Cheddar and various processed cheese slices, were procured from the market. The samples were analyzed for meltability using the tube melt, Schreiber melt, and rheological (G′, G″, and tan δ) tests. The thermal IR image camera test method was developed, and the data were processed using software to determine the melting point. The experiment was conducted in triplicate using different lots of cheese. The collected data were statistically analyzed using RStudio. The melt temperature points from the thermal image camera after 1 min were then correlated with the rheological analysis, and the R^2^ was calculated. The melt characteristics differed significantly according to cheese type. The melting points (tan δ) obtained using a rheometer for cheeses varied between 46.66°C and 64.33°C, and the corresponding values from image analysis were between 48.07°C and 64.61°C. The R^2^ values for the correlations ranged from 0.804 to 0.813, demonstrating a strong relationship between the rheological and image analysis methods. Additionally, thermal images identify the cheese's melting point and heat distribution. The detailed interpretation aids researchers in understanding the thermal properties and structural changes during melting, while also providing sales professionals with informative visual representations to effectively showcase product quality and performance. In conclusion, the study demonstrated that a thermal imaging method provides a practical, quick, and cost-effective approach for estimating the melting point in cheese compared with conventional rheological analysis and can be used on-site.

## Highlights

• The melting characteristics of cheese are crucial for assessing functionality.
• The novel thermal infrared imaging method estimates meltability in real time.
• Strong correlations were found between melt tests and thermal image data.
• Image analysis software provides thermal mapping and heat distribution analysis.
• This rapid method can be used by researchers and by salespeople.

## Graphical Abstract

**Figure:**
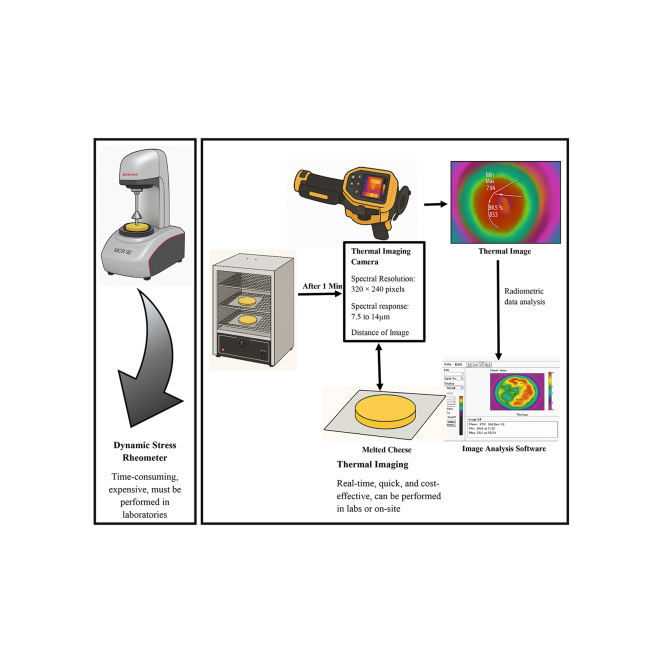
**Summary:** Cheese meltability is a vital parameter for assessing functionality. Various empirical tests are being used to measure cheese's melting point and melting characteristics, but they are time-consuming and must be performed in laboratories. Thermal imaging with an infrared camera is a quick, cost-effective, convenient, and simple method of analyzing the meltability of cheese. The developed test protocol allows this test to be conducted at the sales point or on-site using an infrared camera and inbuilt data analysis, which provides the melting point and heat distribution. The developed test shows high correlation with conventional tests. The test can be performed by any person with little procedural training. The detailed interpretation aids researchers in understanding the thermal properties and structural changes during melting, while also providing sales professionals with informative visual representations to effectively showcase product quality and performance.

Meltability is an important functional characteristic of cheeses and a major selling point in trade, especially for natural cheeses, mozzarella cheese, and processed cheese (**PC**). The industry wants cheese to either melt on pizza or burgers or have a restricted melting point for on-the-go burgers or convenience products. Expectations from every cheese variety are different from a functionality point of view, and hence, the meltability and melting point are important. Natural cheeses and PC are expected to melt, but pasta filata type cheeses (mozzarella or imitation mozzarella cheeses) are expected to melt and stretch (Kapoor and Metzger, 2008; Salunke et al., 2023).

Meltability is a complex functional property that is influenced by various factors. Various methods, including the Schreiber melt test, modified Schreiber melt test (**MSMT**), tube melt test (**TMT**), dynamic stress rheology (**DSR**), or rapid visco analyzer melt test, have been used to study meltability. The ingredients used, especially proteins (intact or unhydrolyzed CN), influence meltability depending on chemistry as well as the thermodynamic properties of cheese (Lucey and Fox, 1993; Johnson, 2000; Lucey et al., 2003; Salunke and Metzger, 2022). The presence of intact CN results in extensive interactions, creating a fibrous CN network; in contrast, the presence of hydrolyzed CN results in weaker interactions, creating a nonfibrous CN network (Garimella Purna et al., 2006), all affecting the meltability of cheese. Melting is primarily determined by the number and strength of the CN-CN interactions (Park et al., 1984). Hydrolysis (because of proteolysis) will break CN bonds, increasing melt but decreasing stretch (Johnson, 2000). Increasing whey protein in cheese decreases meltability (Savello et al., 1989; Salunke and Metzger, 2022). Calcium and the state of calcium in cheese also affect meltability. Hence, aged cheese will melt more easily than young cheese. This variability in cheese poses challenges in the determination of meltability. The MSMT and TMT methods are empirical and are widely used in the industry to measure the meltability of cheese. However, MSMT can result in nonuniform melting due to sample preparation and heating conditions. The TMT method provides quantitative data on melt flow but requires precise temperature control and has a longer test duration, up to 60 min. Hence, determining the exact melting point or melting temperature is a challenge. Currently, DSR is an objective method that provides detailed information on melting behavior and identifies precise melting points. Dynamic stress rheology is used to measure the elastic modulus (G′) and loss modulus (G″) by a temperature sweep (20°C–90°C) test in the viscoelastic region. The crossover point, also known as transition temperature (**TT**) of changeover from solid to liquid cheese, is identified as a loss tangent (tan δ) or transition point where G′ = G″ or tan δ = 1 (Sutheerawattananonda and Bastian, 1998). However, depending on the parameters selected and testing protocol used, the test requires ~2 h per sample, including the cooling period to start a new sample. It also requires specialized equipment and trained personnel, making it unsuitable for on-site assessments. The system is costly and, depending on its configuration, requires a fluid-based temperature control unit, such as a circulating bath with silicon oil connected to a heating jacket. Comparing all the available tests, except DSR, does not give melting point temperatures. All these tests are generally conducted in laboratories and are challenging to conduct on consumer sites. This makes it difficult for the salesperson to demonstrate and sell the cheese.

Thermal infrared (**IR**) imaging is a noninvasive and sophisticated technique that uses IR technology to detect heat emissions by converting IR energy (visible to the human eye) into a visible light display (Fluke, 2025). Thermal imaging is based on the principle that all objects emit IR energy as a function of their temperature, and this energy can be detected and converted into a visual image by the thermal imaging system. The image captured by a thermal image camera can be analyzed and interpreted facilitated by a color palette, where each color represents a specific range of temperatures, for example, shades of blue often indicating cooler temperatures and shades of red representing hotter areas (Fluke, 2025). Thus, a thermal image provides a detailed temperature map of the surveyed area, highlighting hotspots and potential anomalies that may need further investigation. Infrared imaging can offer a novel, real-time approach to observing cheese meltability by capturing thermal variations during heating. It can be used as a continuous monitoring method and integrated into the production lines for real-time assessment (Bagavathiappan et al., 2013). This emerging technology can be used for process optimization as an alternative to conventional laboratory testing (Usamentiaga et al., 2014).

The limitations of traditional methods in terms of time emphasize the need for advanced, real-time techniques, such as thermal imaging, to improve efficiency and applicability in research, industrial, and marketing setups. This study used 2 commercially available natural Cheddar cheeses (mild and extra-sharp) and 4 pasteurized PC slices from 3 different manufacturing lots. These samples were used to evaluate melted functionality and thermal image analysis. The experiment was conducted in triplicate, with each analysis performed in duplicate. Melted characteristics (TMT, MSMT, and DSR) were performed for each sample. Thermal image analysis was conducted following the MSMT, and images were processed using specialized software to determine the melting point. The data collected for TT (°C), image analysis temperature (°C), TMT (mm), and MSMT (mm^2^) among different types of cheeses were statistically analyzed using RStudio (version 4.4.0; https://posit.co/). One-way ANOVA was performed separately for Cheddar and PC slices to assess significant differences within each category. Statistical significance was determined at *P* < 0.05 using Tukey's test.

The MSMT was performed using the method described by Salunke and Metzger (2023) using a disk of cheese samples (2 mm thick × 26-mm diameter) placed on aluminum plates (100 mm × 100 mm, 0.95-mm thick) and heated at 90°C for 7 min in a forced draft oven (Fisher Scientific). The change in the area of the melted cheese in millimeters squared relative to the original area was measured. For the TMT, the procedure described by Modi and Salunke (2024) was used, in which a shredded cheese sample (10 g) was placed in a test tube (29 mm × 20 mm) to make a plug at the bottom and was marked. The test tubes were kept vertically in a refrigerator at 4°C for 30 min. Each test tube was covered with aluminum foil, and small holes were punctured before heating to allow the release of hot gases. After that, they were placed horizontally in the oven (104°C for 60 min). Meltability was measured as the flow distance (mm) of melted cheese from the marked point to the end at which the cheese had stopped flowing.

The dynamic rheological analysis was conducted following the method described by Modi and Salunke (2024). For DSR, cheese slices were cut (2 mm thick × 28.3 mm in diameter) at 20°C, and the analysis was performed using a modular compact rheometer (MCR 92, Anton Paar, Austria) equipped with a 30-mm parallel plate geometry. The exposed edges of samples were coated with mineral oil (paraffin oil, Fisher Scientific) to prevent sample drying during heating measurements. A stress sweep experiment was conducted at a frequency of 1.5 Hz and a constant stress of 100 Pa at 20°C. A temperature sweep (20°C–90°C) was performed using the same rheometer with a constant stress of 100 Pa within the linear viscoelastic region. Measurements were recorded in time sweep mode, with a temperature ramp done at 1°C min^−1^. The elastic modulus (G′) and loss modulus (G″) were determined throughout the experiment. The TT was identified as the temperature at which the G′ and G″ intersected (G′ = G″), corresponding to a loss tangent (tan δ) value of 1 (Biswas et al., 2008). All measurements were performed in duplicate for each sample.

For quick measurement of melting point, thermal imaging was performed on the samples just after the MSMT procedure. However, the developed test can be used soon after the melting of cheese, such as when heating a pizza or burger. The test protocols were developed for the distance and time after which the thermal images were captured. After heating, the sample was removed from the oven and allowed to cool for 1 min before capturing a thermal image using an IR thermal camera (Fluke TiS60+, Fluke Corporation, Everett, WA). The camera parameters included a spectral resolution of 320 × 240 pixels, a spectral response range of 7.5 to 14 µm, and an image acquisition distance of 15 cm. The emissivity was set to 0.95, considering the emissivity of food products. The captured thermal image was analyzed using Fluke Connect software (version 3.5, Fluke Corporation, Everett, WA) to determine the melting point. An ellipse marker was placed on the image to define the region of interest, and a thermal map was generated to extract key temperature parameters, including minimum, maximum, average temperature, and SD. Radiometric data analysis was conducted to quantify the pixel-wise thermal distribution of the sample, providing insights into heat retention and distribution properties. The melting temperature of the sample was calculated based on the extracted thermal data. The R^2^ was calculated to assess the correlation between the melting point obtained via thermal imaging and TT values calculated using the rheometer.

The meltability of the cheese samples was assessed using empirical methods (TMT and MSMT), and an objective (DSR) method, alongside the novel thermal IR imaging technique. The meltability of the cheese samples assessed using the TMT and the MSMT indicated that extra-sharp Cheddar showed significantly (*P* < 0.05) higher meltability (TMT: 90.67 mm; MSMT: 2196.97 mm^2^) than mild Cheddar cheese (TMT: 57.4 mm; MSMT: 1235.93 mm^2^; Table 1). These results suggested that protein matrix structure and aging influence the melting behavior of natural cheeses (Ray et al., 2016; Modi and Salunke, 2024). Conversely, the PC slices displayed significantly lower MSMT values, particularly PC slice-3 (24.82 mm^2^) and PC slice-4 (25.85 mm^2^). A higher stiffness in cheese resulted in a restricted melt and a longer time to reach maximum flow (Lu et al., 2007). The TMT values varied significantly among the PC slices (*P* < 0.05), indicating differences in their meltability. Processed cheese slice-3 showed the highest TMT value, suggesting superior meltability compared with the other PC slices. However, PC slice-2 and PC slice-4 showed significantly lower TMT values, indicating restricted melting. The relationship between TT and cheese meltability (TMT) showed that higher TT values correspond to reduced meltability, particularly in PC slice-1, PC slice-2, and PC slice-3 (Table 1). Garimella Purna et al. (2006) also demonstrated the presence of intact CN in modulating the protein-protein and protein-fat interactions, forming a fibrous CN network, which enhances the cheese matrix's structural integrity and melt resistance.

**Table 1:** Melting characteristics (± SD) of Cheddar and processed cheese slices^1^

Cheese	TT (°C)	Image analysis temperature (°C)	TMT (mm)	MSMT (mm^2^)
Cheddar				
Mild Cheddar	54.33 ± 1.30^a^	54.82 ± 0.31^a^	57.40 ± 4.83^b^	1,235.93 ± 347.42^b^
Extra-sharp Cheddar	55.83 ± 6.61^a^	56.40 ± 0.10^a^	90.67 ± 5.63^a^	2,197.00 ± 105.70^a^
PC				
PC slice-1	64.33 ± 0.76^a^	64.61 ± 0.33^a^	40.98 ± 0.99^b^	28.05 ± 0.95^a^
PC slice-2	60.50 ± 1.32^b^	61.28 ± 0.27^b^	20.41 ± 0.71^c^	28.33 ± 0.37^a^
PC slice-3	46.66 ± 0.58^c^	48.10 ± 0.52^c^	88.53 ± 3.83^a^	24.82 ± 0.15^b^
PC slice-4	61.00 ± 0.50^b^	61.42 ± 0.36^b^	18.27 ± 3.43^c^	25.85 ± 0.40^b^

a–c Values with different superscript letters within the same column differ significantly (*P* < 0.05) within each category.

1 Statistical analysis was performed separately for Cheddar and processed cheese (PC).

The TT of all samples was determined after measuring the G′ and G″ and the results were compared with thermal image analysis. In all samples, G′ values were consistently higher than G″ values, indicating a dominant elastic nature measured across a temperature range of 20°C to 90°C. Natural Cheddar cheeses exhibited stronger gel networks due to their protein matrix and structural integrity, with extra-sharp Cheddar showing the highest values, followed by mild Cheddar (Lucey et al., 2003). In contrast, PC slice samples displayed significantly lower moduli, highlighting the influence of protein composition, moisture content, and fat-protein interactions on their rheological properties. However, the G′ and G″ values of PC samples varied significantly due to the selection of different raw ingredients for PC slice manufacture, which also contributed to differences in TT of all cheeses (Table 1). Gunasekaran and Ak (2003) highlighted that the rheological properties of PC are highly influenced by its protein matrix, moisture, and fat-protein interactions.

Thermal imaging analysis provided the color gradient, with warmer hues representing the sample and cooler hues at the periphery corresponding to the aluminum plate surface temperature (Figure 1). This transition indicated thermal distribution across the samples. The thermal imaging indicated that natural cheeses, such as mild Cheddar and extra-sharp Cheddar, exhibited a more uniform heat distribution, with maximum temperatures closely aligning with their respective TT (Figure 1a and Figure 1b). In contrast, PC samples displayed more varied temperature gradients, which may be attributed to differences in formulation, moisture content, and emulsifier levels affecting heat retention and dissipation. Processed cheese slice-1 (Figure 1c) and PC slice-2 (Figure 1d) showed the highest melting temperature, potentially due to compositional differences. Because the photographs show the quality of melt as well as heat distribution, the thermal imaging test provides additional information that can affect properties like stretchability. After that, the radiometric analysis was performed using the software analysis, which provided the precise melting point temperature.

**Figure 1:**
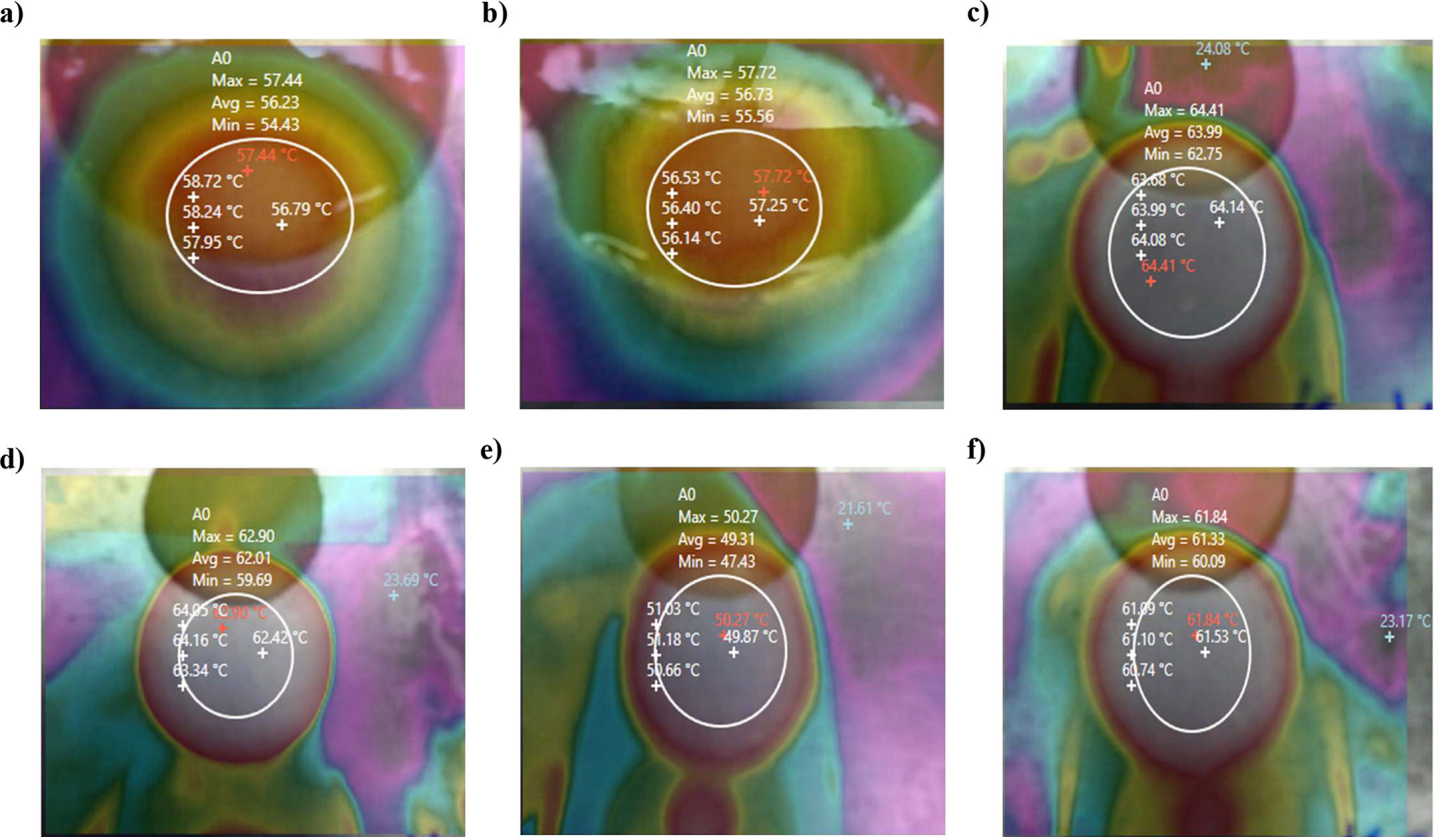
Temperature and heat distribution of Cheddar cheeses and pasteurized processed cheese slices using thermal image camera: (a) mild Cheddar cheese, (b) extra-sharp Cheddar cheese, (c) PC slice-1, (d) PC slice-2, (e) PC slice-3, and (f) PC slice-4. Thermal images of cheese samples after melting, captured using an infrared (IR) camera, show the elliptical region of interest (ROI) marked to analyze the surface temperature distribution.

As presented in Table 1, the results indicated a strong correlation between TT obtained through the rheometer and the image analysis-derived temperature values across different cheese types. For mild and extra-sharp Cheddar, the TT values obtained via rheometer were 54.33°C and 55.83°C, respectively, which were closely aligned with the image analysis results of 54.82°C and 56.35°C (Table 1). This suggests that thermal imaging provides a reliable estimation of the transition phase in natural cheeses. Additionally, it shows the heat distribution as well as the quality of the melt. The minimum variation between the 2 techniques (ΔT ≤0.52°C) showed the robustness of the image-based approach in detecting cheese TT. However, greater deviations were observed in PC slices, where PC slice-3 exhibited a TT of 46.66°C via rheometer but was recorded at 48.07°C through image analysis, with a difference of 1.41°C. Similarly, PC slice-1 demonstrated a small deviation, with TT values of 64.33°C and 64.61°C (image analysis; Table 1). These variations may be attributed to differences in maturity levels of Cheddar cheeses in the PC formulation, emulsifying salts, and moisture content, which affect heat distribution and structural transformation during heating (Modi and Salunke, 2024).

An R^2^ test was performed to validate the radiometric analysis of melting point readings. The melting point obtained from thermal image analysis was compared with the TT measured using a rheometer. The R^2^ values, shown in Figure 2a–f indicated a strong correlation between estimated TT using a rheometer and actual melting temperature derived from image analysis across all cheese samples. The R^2^ values for the correlations ranged from 0.804 to 0.813, suggesting a reliable prediction of TT, although slight deviations exist due to variations in the melted characteristics of the samples.

**Figure 2:**
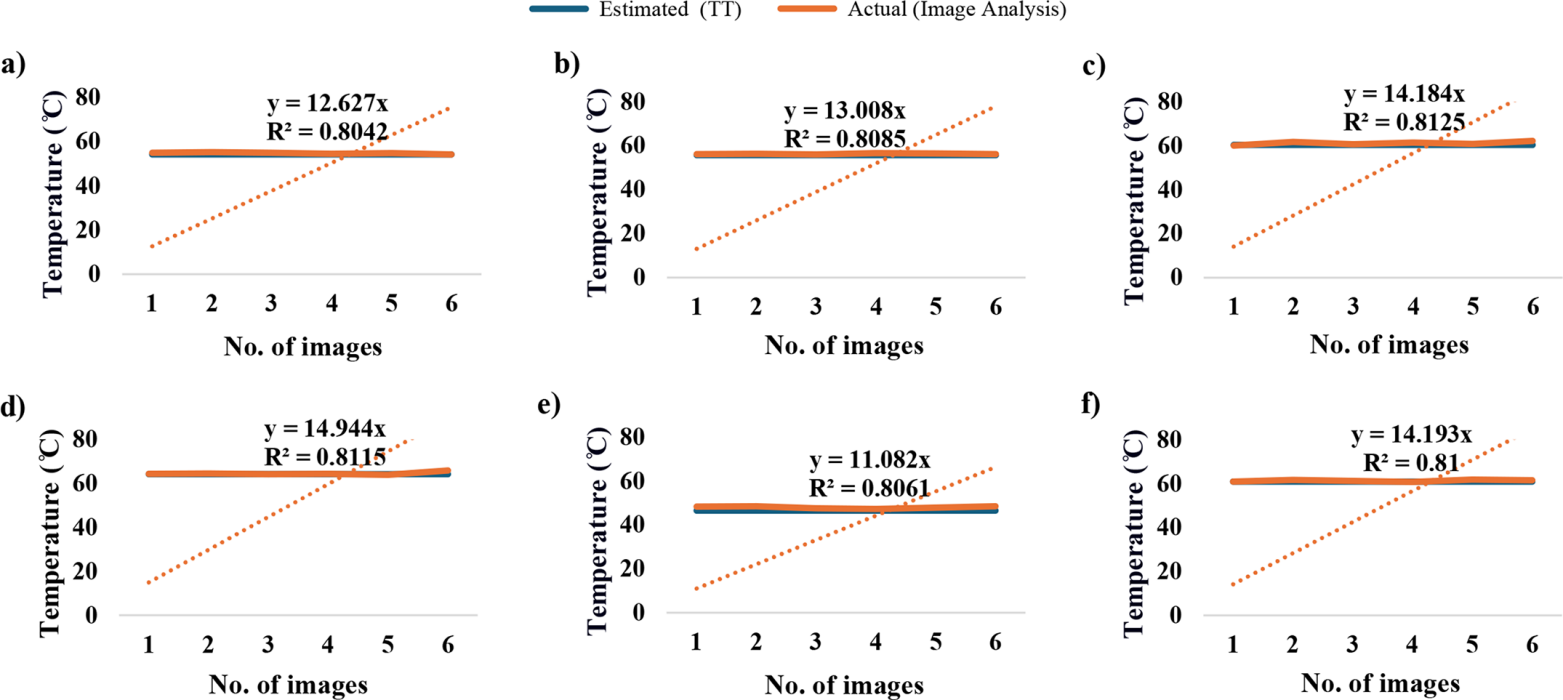
Coefficient of determination (R^2^) between TT and actual (image analysis) temperature of Cheddar cheeses and pasteurized PC slices using a thermal image camera: (a) mild Cheddar cheese, (b) extra-sharp Cheddar cheese, (c) PC slice-1, (d) PC slice-2, (e) PC slice-3, and (f) PC slice-4. The dotted line represents the linear regression between actual (image analysis) and estimated (TT) temperatures.

The current study focused exclusively on Cheddar cheese and PC slices; however, other cheese varieties, such as mozzarella, Cheddar, Swiss, Monterey Jack, and Parmesan, differ significantly in their processing methods, compositions, structural and melting characteristics, and need to be studied. Thermal infrared imaging relies on color gradients to represent temperature variations, and these differences may influence the accuracy and reproducibility of the method. Therefore, extending the application of thermal IR imaging to a broader range of cheese types is necessary to validate its versatility and effectiveness. In addition to that, thermal IR camera calibration can influence the accuracy and consistency of the color representations. Variations in these parameters may lead to changes in temperature readings and affect the reproducibility of results.

Overall, the rheological parameters indicated differences in the TT values of different cheeses, indicating variations in melting behavior. The proposed thermal imaging method significantly reduced the analysis time from almost 2 h to 8 min, offering a rapid and on-site alternative to conventional rheological methods. This approach eliminates the need for specialized laboratory equipment, making it highly practical for researchers and industry professionals. The test is easy to perform using a thermal IR camera and can be carried out on-site. The test not only gives a melting point but also gives an idea about melt quality and heat distribution, which can be correlated with other properties. This technique can also be further applied to other dairy and food products for which heating and temperatures are critical. In conclusion, thermal imaging presents a cost-effective, efficient, and accessible method for real-time estimation of the melting behavior and temperature measurement of cheese, making it a valuable tool for quality assessment in research and industrial applications.
